# Enhancing biological nitrogen fixation in rice paddy ecosystems: challenges, opportunities, and sustainable strategies

**DOI:** 10.3389/fmicb.2026.1714909

**Published:** 2026-02-13

**Authors:** Yanhui Zhang, Haihou Wang, Peifeng Chen

**Affiliations:** 1 Institute of Agricultural Sciences in Taihu Lake District, Suzhou Academy of Agricultural Sciences, Suzhou, China; 2 Suzhou Field Scientific Observation and Research Station for Soil Quality, Ministry of Agriculture and Rural Affairs (MARA), Suzhou, China; 3 Suzhou Key Laboratory of Cultivated Land Quality Monitoring and Conservation, Suzhou, China

**Keywords:** ^15^N₂ labeling, biological nitrogen fixation, diazotrophs, rice paddy ecosystems, sustainable agriculture

## Abstract

Synthetic nitrogen (N) fertilizers have substantially increased rice (*Oryza sativa*) yields but at the expense of low N use efficiency, significant environmental losses, and deterioration of soil health. Biological N fixation (BNF) offers a sustainable and complementary N source, providing a gradual and plant-synchronized N supply that can partially substitute for synthetic N fertilizers. Enhancing BNF in paddy fields to reduce fertilizer inputs has therefore become a topic of considerable scientific and practical interest. This review synthesizes current knowledge of BNF in rice systems, with emphasis on methods for quantifying BNF rates, the ecological and agronomic factors that regulate its magnitude, and the influence of field management practices. It further highlights key challenges, including the inhibitory effects of synthetic N fertilizers on BNF, that constrain the full realization of BNF potential. And it proposes possible solutions such as straw incorporation, the selection and cultivation of ammonium-tolerant diazotrophs, and the application of genetic engineering to develop ammonium-excreting N-fixing bacteria. Collectively, these insights provide a foundation for advancing low-input and environmentally sustainable rice production systems.

## 1 Introduction

Nitrogen (N) is a key determinant of rice (*Oryza sativa*) productivity, underpinning processes such as chlorophyll biosynthesis, amino acid and protein formation, and ultimately grain yield. To meet the escalating global demand for rice, modern production systems have become heavily dependent on synthetic N fertilizers. Current estimates indicate that global N fertilizer use surpasses 100 million tons annually, with rice-based agroecosystems in Asia accounting for a substantial fraction of this consumption (Lee, 2021). Despite such intensive inputs, N use efficiency (NUE) in rice systems typically remains below 50%, implying that more than half of the applied N is lost through leaching, volatilization, or denitrification (Lee, 2021)). These inefficiencies impose economic costs on farmers and exacerbate ecological risks, including groundwater contamination, eutrophication of aquatic ecosystems, and emissions of nitrous oxide (N₂O), a potent greenhouse gas. Long-term over application of synthetic N further degrades soil health, contributing to acidification, depletion of organic matter, and disruptions in microbial community composition, ultimately reducing biodiversity and increasing vulnerability to biotic stresses (Wang et al., 2022; Koushal et al., 2025). Against this backdrop, the development of alternative strategies for sustainable N management in rice agroecosystems has become an urgent priority.

One promising avenue lies in harnessing biological N fixation (BNF), whereby diazotrophic microorganisms reduce atmospheric N₂ into plant-available ammonium. The flooded, carbon-rich conditions of paddy soils create favorable ecological niches for diazotrophs (Koushal et al., 2025; Chen et al., 2025). Historically, BNF has been recognized as a central mechanism enabling rice cultivation over centuries in systems with minimal or no synthetic fertilizer input. Recent evidence demonstrates that microbially mediated N₂ fixation is widespread in rice paddies and contributes substantially to soil fertility under flooded conditions (Yoshida and Ancajas, 1971; Ladha et al., 2022). A number of studies have shown that N-fixing activity is substantially greater in paddy soils than in the soils of other cropping systems (Yoshida and Ancajas, 1973; Ladha et al., 2016), which helps explain why the subsistence agriculture of the pre-chemical era was able to effectively preserve the N status of paddy soils by balancing N loss through grain harvest with N gain from BNF (Ladha and Reddy, 2003).

Nevertheless, BNF is highly sensitive to external N inputs, with synthetic fertilizers exerting strong suppressive effects (Reed et al., 2011; Vitousek et al., 2013). BNF activity declines by more than 80% when application rates exceed 125 kg N ha^−1^ (Zhang Y. H. et al., 2021, Zhang Z. B. et al., 2021), and remains markedly inhibited even at relatively low inputs of around 80 kg N ha^−1^. Yet, to sustain high yields, N inputs in Asian paddy systems commonly exceed 200 kg N ha^−1^ (Zhang et al., 2013). Although tropical lowland rice can achieve yields of 2–3.5 t ha^−1^ through reliance on soil N mineralization and diazotroph-mediated BNF (Ladha and Reddy, 2003), such yields are insufficient to ensure food security in densely populated regions such as Southeast Asia. This creates a central paradox: while chemical N fertilizers remain indispensable for yield targets, their application undermines the very biological processes that could mitigate over dependence on them. This paradox motivates further investigation into how BNF can be sustained under fertilized conditions.

To move beyond this paradox and advance toward BNF-sustainable fertilization, an integrative framework is required, one that explicitly links quantitative constraints, microbial ecological mechanisms, and agronomic management rather than treating them in isolation. Recent advances in *in situ* ^15^N_2_ labeling provide direct, field-based estimates of BNF rates and their spatial variability, establishing realistic benchmarks and suppression thresholds under fertilized conditions (Reed et al., 2011; Ma et al., 2019a; Wang X. J. et al., 2020; Zhang Y. H. et al., 2021). At the same time, molecular and isotopic approaches (e.g., nifH sequencing and ^15^N-SIP) are revealing how diazotroph diversity, community assembly, and functional persistence respond to mineral N inputs and other environmental filters, helping to explain why certain taxa or consortia retain activity under moderate fertilization (Xiong et al., 2024; Ma et al., 2019b; Wang X. J. et al., 2020). These ecological insights, when interpreted alongside agronomic studies defining fertilizer regimes, timing, residue management, and water control, clarify how chronic N suppression emerges, and how it may be partially alleviated without sacrificing yield (Zhang et al., 2013; Zhang et al., 2022). Bridging these three strands reframes the apparent conflict between yield and biology as an optimization problem, in which BNF can potentially be sustained, targeted, and enhanced within realistic fertilization regimes rather than excluded by them.

This review therefore aims to bridge three currently fragmented areas: (i) quantitative evidence from *in situ* ^15^N_2_ labeling, (ii) emerging insights into diazotroph diversity and community assembly in paddy soils, and (iii) the paradox that high mineral N inputs, while agronomically indispensable, chronically suppress BNF. By synthesizing these strands, we aim to elucidate why BNF in many modern paddy systems remains below its theoretical potential and to outline management and biotechnological strategies to enhance BNF under realistic fertilization regimes.

## 2 The paddy soil environment and its diazotrophic communities

**Figure 1:**
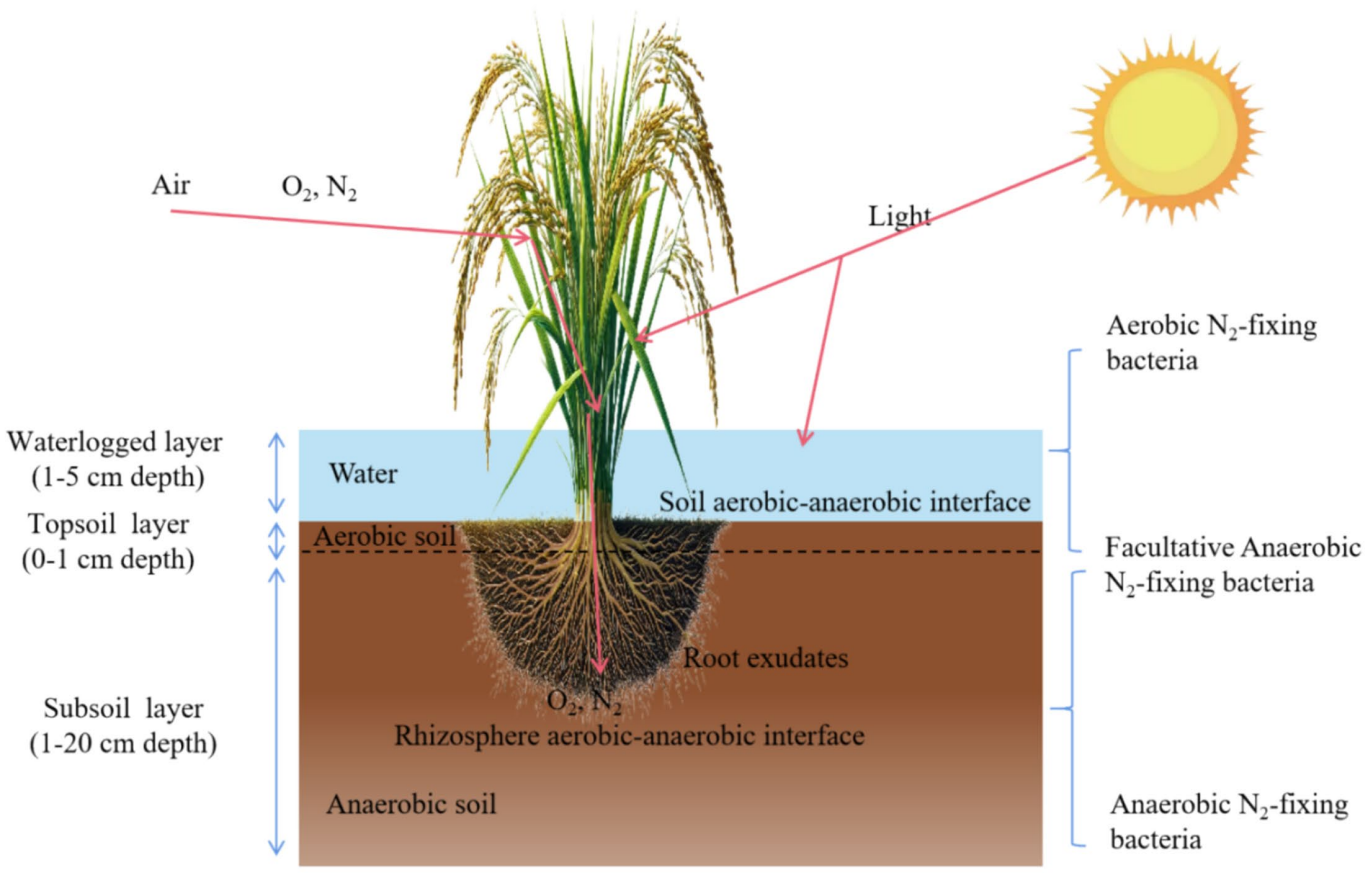
Schematic diagram of a flooded paddy system and N-fixing microorganisms.

Understanding BNF in rice paddies requires first recognizing the unique environmental heterogeneity of flooded soils and the ecological organization of their diazotrophic communities. Flooded rice fields provide unique advantages for rice cultivation, such as continuous water supply, gradual neutralization/acidification of soil pH, and enhanced BNF. In subsistence agriculture without N fertilizers, lowland rice generally outperforms upland crops in terms of yield, with stable productivity and maintained soil N fertility attributed to BNF (Roger and Watanabe, 1996; Ladha and Reddy, 2003). Flooding creates anaerobic conditions in the reduced soil layer just below the surface, resulting in five distinct BNF environments in rice-cropped, flooded soils, influenced by physicochemical properties, light, and energy supply (Roger and Watanabe, 1996). These include (Figure 1): (1) In the photic, aerobic floodwater layer, organic matter and nutrient cycling are driven by a community of producers (e.g., algae, weeds) and consumers (e.g., bacteria, zooplankton), in addition to supporting symbiotic systems like Azolla-Anabaena; (2) the thin, photic, oxidized surface soil layer with a positive redox potential, harboring cyanobacteria and aerobic bacteria; (3) the non-photic, anaerobic reduced soil layer with a negative redox potential, where microbial activity concentrates in organic detritus-containing aggregates; (4) the rice plant itself, which provides a habitat for epiphytic bacteria and algae and a rhizosphere environment that supports microbial growth; and (5) the plow sole layer, which is aerobic in well-drained soils and anaerobic in poorly drained soils, with significant N-supplying microbial activity at its surface (Yoneyama et al., 1977; Ventura and Watanabe, 1984). These diverse environments contribute significantly to BNF and nutrient cycling in flooded rice systems.

N-fixing microorganisms, including symbiotic, associative, and free-living groups, occur in multiple niches of the rice paddy system. These microorganisms have been detected in rice seeds, plant tissues, rhizosphere soil and bulk soil. Numerous studies using *nifH* PCR-DGGE, high-throughput sequencing, and gene expression assays (e.g., RT-PCR) have revealed that diazotrophic communities in paddy fields are taxonomically heterogenous, and that different taxonomic groups may dominate under different environmental conditions (Lotta et al., 2009). Symbiotic systems (e.g., Azolla-Anabaena) coexist with associative and free-living N_2_ fixers, and recent reviews emphasize that plant-associated diazotrophs, particularly those linked to rice roots, are often the dominant BNF agents in intensively cropped wet rice (Pittol et al., 2015). Culture-dependent isolation and nifH-targeted molecular surveys have revealed diverse diazotrophic communities in the rice rhizosphere and bulk paddy soil, including iron-reducing genera such as *Anaeromyxobacter* and *Geobacter*, which can predominate under flooded, reduced conditions (Masuda et al., 2023; Zhang Z. C. et al., 2023). However, surface soils host abundant cyanobacterial diazotrophs (Wang et al., 2019; Ma et al., 2019a,b; Wang J. L. et al., 2020), whose quantitative contribution is addressed in Section 4. Parallel work on plant tissues has shown that endophytic N-fixing bacteria colonize rice roots, stems and leaves, and that their activity and community structure vary among genotypes and developmental stages, indicating a regulated, functional association with the host (Ueda et al., 1995; You et al., 2005; Prakamhang et al., 2009; Rangjaroen et al., 2014). Moreover, surface-sterilized seeds of cultivated and wild rice harbor cultivable and non-cultivable endophytic bacteria, some with demonstrated plant-growth-promoting and putative diazotrophic traits, supporting the view that seedborne communities act as an inoculum reservoir for subsequent root and rhizosphere assemblages (Zhang Z. B. et al., 2021). These habitats collectively support diverse symbiotic, associative, and free-living diazotrophs in rice paddies.

The functional importance of diazotroph diversity and community composition becomes evident when linking community structure to BNF potential. A study across a climatic gradient (mid-temperate, warm-temperate, subtropical, tropical) in eastern China found that diazotroph diversity (measured via *nifH* sequencing) was significantly higher in tropical paddy soils, and lowest in mid-temperate soils; yet potential nitrogenase activity peaked in warm-temperate zones (Wu et al., 2020). It should be noted that *nifH* gene abundance and potential nitrogenase activity reflect the genetic capacity and short-term enzymatic potential for N₂ fixation, rather than realized N inputs quantified by ^15^N_2_ tracer methods. Multivariate analyses (e.g., canonical correlation, structural equation modeling) suggested that climate and soil nutrients indirectly influenced potential nitrogenase activity through their effects on soil pH and diazotroph community structure, while the community composition itself, along with soil C/P ratio and *nifH* gene abundance, directly affected potential nitrogenase activity (Wu et al., 2020). Interestingly, in a survey covering 76 paddy soils over six major rice-growing regions in China, no significant correlation was found between asymbiotic N fixation rate and *nifH* gene abundance. Instead, environmental factors such as soil molybdenum (Mo), total N, total phosphorus (P), and organic carbon content showed stronger correlations with asymbiotic N fixation rate; whereas *nifH* abundance correlated more with soil C: N ratio, exchangeable calcium (Ca), and pH. This implies that diazotroph diversity and community composition (not just sheer abundance) modulate N fixation efficiency, likely through metabolic specialization and ecological interactions (Hu et al., 2024). Furthermore, agricultural practices and soil management can substantially influence diazotroph community structure and functional outcomes. For example, amendment with biochar under flooding conditions altered community composition (increasing relative abundance of genera such as *Pelomonas* and *Azospirillum*) and increased *nifH* gene abundance, suggesting increased potential for N fixation under these modified conditions (Chen et al., 2024). Similarly, return of crop residues (e.g., straw incorporation) was found to affect *nifH* gene abundance and community composition, though the response of nitrogenase activity was variable and depended on soil type, root-proximity (rhizosphere vs. bulk soil), and residue loading rate (Zhao et al., 2022; He et al., 2023).

In paddy soils, indigenous diazotroph diversity strongly conditions how inoculant strains establish and function. A 76-site soil sample across Chinese rice regions showed that asymbiotic N_2_-fixation rates were best predicted by diazotroph community composition and diversity, together with soil pH and C:N:P stoichiometry, whereas *nifH* gene abundance alone explained little of the variance (Hu et al., 2024). This means that inoculants are introduced into already complex, environmentally filtered assemblages. Community-ecological work further demonstrates that site-specific “microbial species pools” largely determine which diazotrophs can colonize roots and leaves, and that local assembly processes govern coexistence and functional expression (Xiong et al., 2024). In long-term fertilized paddy fields, increasing NPK dose reshapes diazotroph diversity and shifts community assembly from more deterministic to more stochastic processes, thereby altering the niche space available for introduced strains (Wang X. J. et al., 2020). Field inoculation studies illustrate this context dependence: Azospirillum sp. B510 increased rice tiller number and shoot length without becoming abundant itself (<0.5% of the community), instead enriching specific N_2_-fixing and plant-growth-promoting taxa in the rhizosphere and phyllosphere (Yasuda et al., 2022), while cyanobacterial and Calothrix-based biofertilizers significantly restructured rhizosphere and bulk-soil bacterial communities in a soil-type-dependent manner (Ranjan et al., 2016). Together, these findings indicate that effective diazotroph inoculants for paddy systems must be designed with indigenous diazotroph diversity and assembly rules in mind, favoring locally adapted strains or consortia that integrate into resident communities rather than attempting to override them.

These findings underline that diazotroph diversity and community composition in paddy soils are not static or uniform, they vary spatially and temporally under the influence of climate, soil physicochemical properties, and management practices. More importantly, such variation in community structure has functional consequences: different communities exhibit differing capacities for BNF, potentially modulating N inputs and thereby influencing soil fertility and crop yield. Thus, maintaining or managing for high diazotroph diversity (or optimal community composition) might be a promising strategy for sustainable rice agriculture, especially aiming to reduce synthetic N fertilizer use and mitigate associated environmental problems. Despite these advances, several knowledge gaps remain. First, the relative contributions of different diazotroph groups (e.g., cyanobacteria, heterotrophic bacteria, iron-reducing bacteria) to total BNF in different paddy environments remain uncertain, particularly under varying water regimes, nutrient management, and soil types. Second, the ecological mechanisms, e.g., competition, niche partitioning, syntrophic relationships, that shape diazotroph community assembly and mediate function are still poorly understood. Third, long-term field studies coupling high-resolution community profiling (e.g., metagenomics or metatranscriptomics) with direct measurements of N fixation (e.g., ^15^N-tracing) under realistic agricultural management are scarce. Addressing these gaps will be essential to harness the potential of diazotrophic communities for sustainable agricultural N management.

Collectively, the spatial heterogeneity of paddy soil environments and the diversity and assembly of diazotrophic communities define the ecological boundaries within which BNF operates. These features underpin where BNF occurs, which microbial groups contribute, and how fixation responds to mineral N inputs. As such, this section provides the conceptual foundation for interpreting methodological estimates of BNF (Sections 3–4) and for evaluating agronomic and biotechnological strategies aimed at sustaining BNF under fertilized conditions.

## 3 The measurement methods of BNF in rice paddies

To study BNF, it is essential to determine the BNF rate. Importantly, differences among BNF quantification methods are not merely technical details but have profound implications for reported fixation rates and inferred spatial patterns. Variability in conversion factors, system enclosure effects, and temporal integration can lead to divergent estimates of BNF across studies. These methodological constraints must therefore be considered explicitly when synthesizing reported BNF magnitudes and identifying fixation hotspots, as discussed in the following section.

Depending on distinct principles and assumptions, there are five distinct methodologies are available for quantifying BNF, and these methods can be broadly categorized as indirect or direct quantification approaches. The most commonly used indirect method for measuring BNF in paddy soil is the acetylene reduction method (Bergersen, 1970; Mariotti, 1983). Other indirect methods include the natural abundance ^15^N method (Bremer and Kessel, 1990), the ^15^N isotope dilution method (Mariotti, 1983), and the N balance method (Giller et al., 1984), among others. The uncertainties associated with these indirect methods are inherent to their distinct principles and assumptions. Key sources of variation include the reliance on inaccurate conversion factors or the challenge of adequately accounting for various N sources and loss pathways. The most direct method for determining BNF is the ^15^N_2_ gas labeling method. This method does not require conversion factors and is not affected by external N sources, allowing for accurate BNF measurement (Zhu et al., 1986). However, the challenge lies in maintaining the system’s airtightness for long periods and ensuring that the environmental conditions in growth chambers (such as CO_2_ concentration, oxygen levels, temperature, and light intensity) closely resemble the field environment for rice growth. Additionally, the high cost of high-purity, high-abundance ^15^N_2_ gas limits the widespread application of this method under natural environmental conditions (Unkovich et al., 2008) (Table 1).

**Table 1:** Methods for measuring BNF in rice paddies.

Method	Principle	Advantages	Limitations	Uncertainty sources and magnitude	References
Acetylene reduction assay (ARA)	Nitrogenase reduces acetylene (C₂H₂) to ethylene (C₂H₄). Ethylene production measured by gas chromatography estimates N₂ fixation.	Low cost; high sensitivity; simple protocol	In-situ incubation disturbs soil; controversial conversion ratio (3:1 or 4:1); high C₂H₂ inhibits nitrogenase	High uncertainty. Major sources include variable C₂H₂ → N₂ conversion factors, inhibition of nitrogenase activity by acetylene, disturbance of soil redox conditions, and temporal variability of nitrogenase activity.	Bergersen (1970) and Mariotti (1983)
^15^N isotope dilution	^15^N-labeled fertilizer added to soil. N₂-fixing plants incorporate unlabeled N₂, lowering ^15^N abundance compared to non-fixing plants.	Reflects field conditions; high accuracy	Long-term trials; expensive; depends on reference plant selection	Moderate uncertainty. Uncertainty arises from non-uniform fertilizer distribution, differential root uptake, N recycling, and inappropriate reference plant selection.	Mariotti (1983)
Natural ^15^N abundance (δ^15^N)	N₂-fixing plants have δ^15^N values near atmospheric N₂ (~0‰), while non-fixers show higher δ^15^N (soil N enriches ^15^N).	Non-destructive; no tracer addition	Requires precise background δ^15^N; high spatial variability in soil interferes	Moderate to high uncertainty. Sensitive to spatial heterogeneity in soil δ^15^N, fractionation during N transformations, and low BNF signal strength. Small analytical errors can cause large proportional errors, especially when BNF contribution is low.	Bremer and Kessel (1990)
N difference method	Compares total N accumulation between N₂-fixing and non-fixing plants.	Field-adaptable; no specialized equipment	Ignores soil N mineralization differences; underestimates BNF	High uncertainty. Cannot separate BNF from soil N mineralization or deposition; highly sensitive to soil fertility gradients and management history.	Giller et al. (1984)
^15^N₂ gas tracer (^15^N₂ direct labeling method)	Direct injection of ^15^N-labeled N₂ gas into the system. ^15^N enrichment in plants/microbes quantifies fixation.	Most direct; high precision	Complex setup; very expensive; hard to simulate natural conditions	Low uncertainty. No conversion factors required and independent of external N sources. Main uncertainties relate to system airtightness, gas equilibration, and representativeness of environmental conditions.	Unkovich et al. (2008) and Bei et al. (2013)

**Figure 2:**
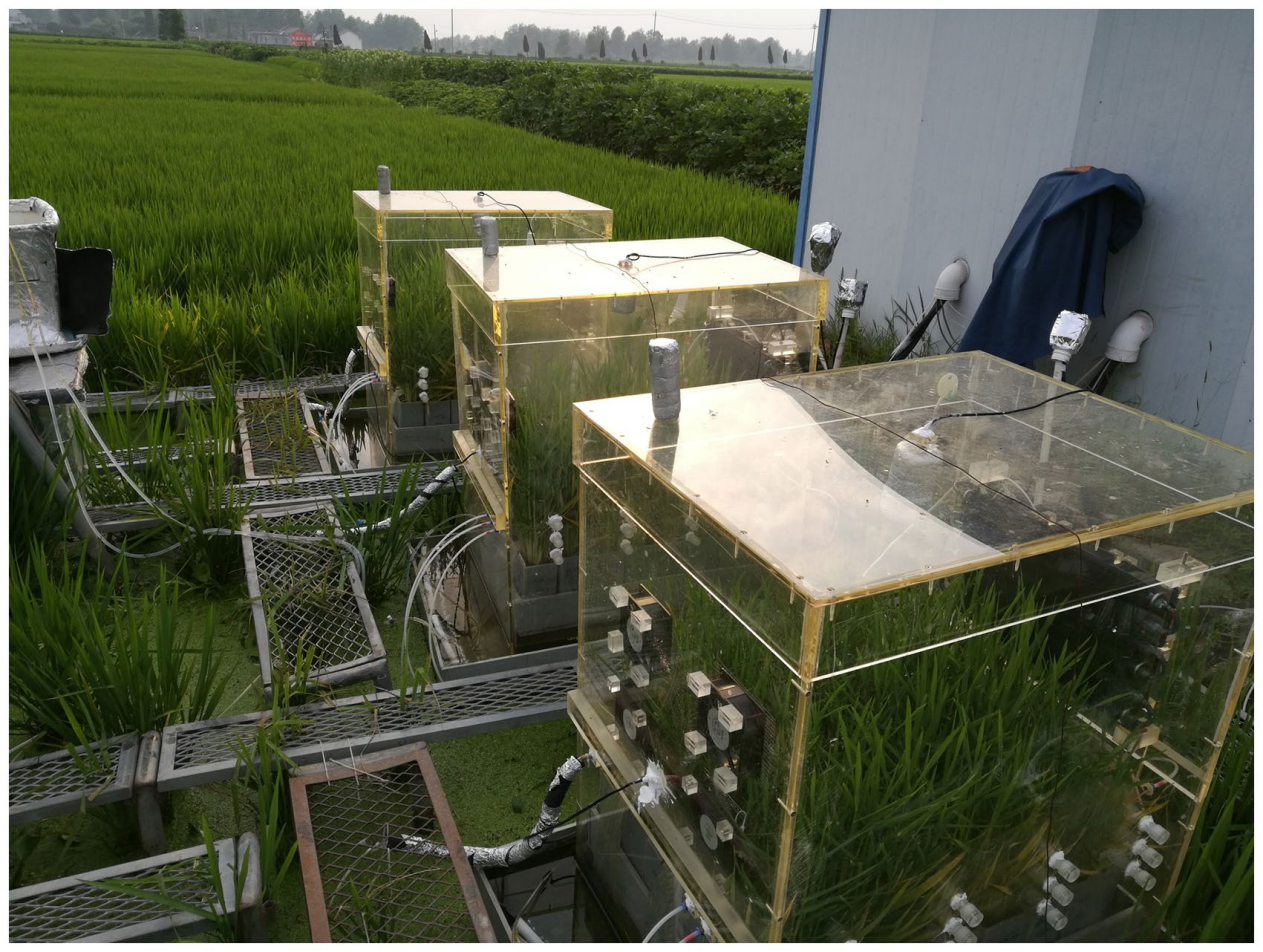
In-situ field air-proof plant growth control system (Zhang Y. H. et al., 2021;Zhang Z. B. et al., 2021).

In China, Bei and Xie developed an in-situ intelligent airtight plant growth control system for field applications (Figure 2), which has been successfully applied in BNF research (Bei et al., 2013). This system automatically regulates internal temperature (outside temperature ± 1 °C) and CO₂ concentration (outside CO₂ concentration ± 20 ppm) while maintaining high airtightness. After iterative upgrades, this system has been enhanced to regulate water levels, apply pesticides, and maintain oxygen concentrations, enabling highly accurate quantification of BNF throughout the whole rice growth season (Figure 1). Using this ^15^N_2_ labeling system, it is usually labeled over the entire rice growing season, the team conducted extensive research on BNF rates and their hotspot distribution in paddy fields (Bei et al., 2013; Ma et al., 2019a,b; Wang et al., 2019; Wang X. J. et al., 2020; Zhang Y. H. et al., 2021; Zhang et al., 2022, 2025; Hu et al., 2024). The findings will be discussed together with results from other studies in the following section. It should be emphasized, however, that while such chamber-based systems provide high process-level accuracy for quantifying BNF under controlled *in situ* conditions, they do not fully capture ecosystem-scale representativeness. Enclosure inevitably alters gas diffusion, microclimate, and potentially plant-microbe interactions compared with open-field conditions. Therefore, results derived from this system are most appropriately interpreted as robust estimates of BNF processes and relative spatial patterns (e.g., vertical hotspots), rather than as direct proxies for whole-field BNF budgets. At the same time, since this system is completely airtight and thus measures the total amount of N fixed over the entire rice-growing season, its results cannot reflect the temporal variation pattern of BNF (e.g., across different growth stages of rice or diurnal cycles). Therefore, future studies need to integrate long-term and multi-temporal observations to better capture the dynamic variation of N fixation under real field conditions and to assess the uncertainty when extrapolating results from closed-chamber measurements to the regional scale.

## 4 Amount and spatial hotspots of BNF in paddy fields

BNF in paddy soils is spatially heterogeneous and vertically stratified, reflecting strong gradients in light availability, redox conditions, substrate supply, and microbial community composition. The dominant BNF systems include phototrophic diazotrophs (mainly cyanobacteria) inhabiting floodwater and surface soil, together with heterotrophic and iron-reducing diazotrophs located in the rhizosphere and reduced subsoil (Ladha and Reddy, 2003). And BNF is contributed roughly equally by phototrophic and heterotrophic diazotrophs (Bei et al., 2013). Early estimates based on acetylene reduction assays (ARA) and N balance approaches suggested that seasonal BNF in unfertilized rice paddies could reach 40–60 kg N ha^−1^, with cyanobacteria and heterotrophic bacteria contributing comparable fractions (Roger and Ladha, 1992; App et al., 1984), it should be noted that these early estimates, derived primarily from acetylene reduction assays or N balance methods, are associated with higher uncertainty due to methodological assumptions. However, recent advances in *in situ* ^15^N₂ labeling have generally reported lower fixation rates, typically ranging from 1 to ~22 kg N ha^−1^ in unfertilized systems (Ma et al., 2019a; Wang et al., 2019; Hu et al., 2024). These discrepancies likely reflect methodological differences outlined in Section 3 as well as legacy effects of long-term fertilization, although the relative importance of these factors remains insufficiently resolved. Distinguishing between these causes carries significant implications. If the variation arises primarily from improved measurement accuracy, the currently reported, relatively low BNF rates may represent a more realistic ceiling of the natural potential. If, however, it reflects a genuine decline in BNF capacity due to fertilization-induced changes in soil properties, then investigating strategies to restore or enhance this capacity becomes compelling, both for ecological sustainability and potential long-term economic benefit.

**Figure 3:**
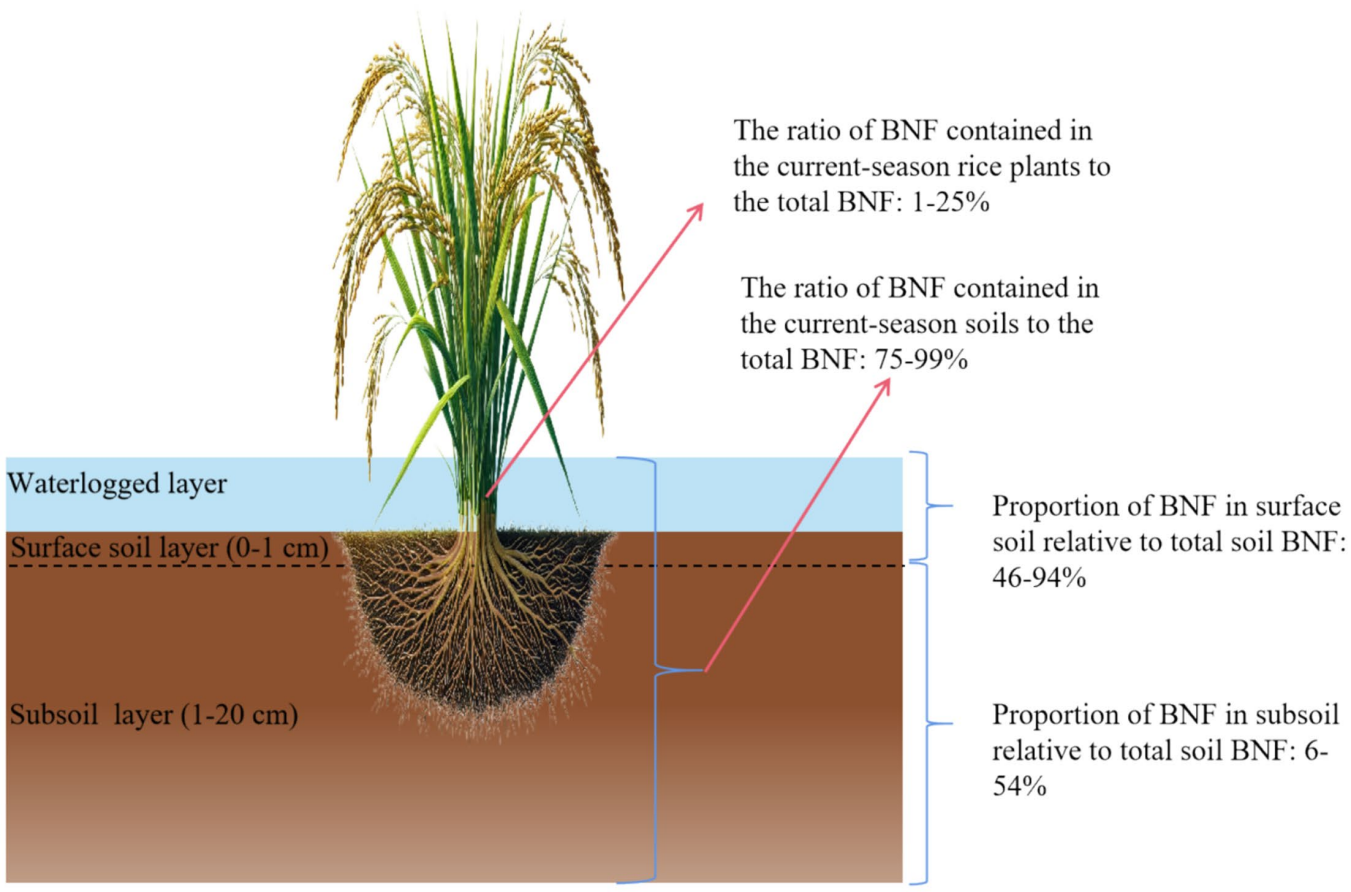
Schematic distribution of BNF in current-season rice plants and soil, and its partitioning between surface (0–1 cm) and subsoil layers (1–20 cm).

Identifying and understanding hotspots of BNF is critical for both mechanistic research and management (Figure 3). Based on in situ ^15^N_2_ tracer experiments, which directly track newly fixed N, field experiments in paddy microcosms have shown that newly fixed N is strongly enriched at the water-soil interface: in a flooded paddy system, 55% of the fixed ^15^N associated with soil was recovered in the surface soil layer (0–1 cm), where phototrophic *cyanobacteria* and other surface biofilms dominate (Bei et al., 2013). More recent combinations of chamber-based ^15^N_2_-labeling, ^15^N-DNA-SIP and NanoSIMS confirm that 94.08% of biologically fixed N in the soil can be recovered in the 0–0.5 cm soil layer, and that *Nostocales* and *Stigonematales* cyanobacteria are the major active diazotrophs in this illuminated surface niche (Wang X. J. et al., 2020). A synthesis of multiple studies indicates that 46–94% (46, 55, 60, 70, 86, 94%) of BNF in the 0–1 cm soil layer, whereas only 6–54% occurs in the subsoil (Bei et al., 2013; Wang et al., 2019; Ma et al., 2019a; Zhang Y. H. et al., 2021; Wang T. et al., 2024). Given that these trials were conducted in different years while employing the same soil type and identical methods (^15^N_2_ gas tracer) for measuring BNF, the considerable variation in the proportion of N fixation contributed by the 0–1 cm soil layer may be attributed to differences in temperature and light conditions across years. This is particularly relevant because surface-layer photosynthetic diazotrophs are highly sensitive to both temperature and light (Kondo and Yasuda, 2003; Brauer et al., 2013). However, this hypothesis still requires experimental verification. Below the surface, the rhizosphere and reduced subsoil also act as important hotspots for heterotrophic and iron-reducing diazotrophs. Recent SIP-based studies identify *Anaeromyxobacter* and *Geobacter* as dominant active N_2_-fixers in anoxic, Fe-reducing horizons, whose activity can be further stimulated by Fe amendments (Masuda et al., 2023). However, the amount of BNF utilized by the current-season rice crop (including both endophytic fixation and fixed N absorbed from the soil) accounts for only 1–25% of the total fixed N, with one study reporting a higher value of 47% (Bei et al., 2013), while the remaining 75–99% is retained in the soil (Wang T. et al., 2024). This indicates that endophytic diazotrophs are not the primary contributor to BNF in paddy fields. In addition, the transfer rate of fixed N from the microbial pool to rice plants is primarily controlled by microbial turnover rates, the timing of organic N mineralization, and the synchronization between N release and crop N demand (Schimel and Bennett, 2004; Kuzyakov and Blagodatskaya, 2015). Rapid microbial immobilization and slow biomass turnover can sequester a large fraction of fixed N in microbial and soil organic pools, whereas delayed mineralization often leads to mismatches with peak plant N uptake, limiting agronomic benefits (Manzoni et al., 2012). In contrast, accelerated microbial turnover and rhizosphere-driven mineralization can enhance the availability of fixed N to crops. Nevertheless, how microbial turnover, organic N mineralization, and crop-microbe synchronization jointly govern the availability of fixed N to rice plants under field conditions remains unclear and requires further study. Consequently, assessing the contribution of BNF to the current-season crop requires not only quantifying the total amount of N fixed but also understanding its release dynamics. Optimizing management practices (e.g., organic amendment input, water regulation) to promote the release of fixed N during key crop N demand stages is crucial for maximizing the agronomic value of BNF.

It should be emphasized that these quantitative hotspot estimates are primarily derived from chamber-based *in situ* ^15^N_2_ labeling studies conducted in continuously flooded paddy soils in China. Their representativeness across different rice-growing countries and regions, soil textures, fertilization histories, and water regimes remains uncertain. Extrapolation of surface-dominated BNF patterns beyond East Asian flooded paddies should therefore be made with caution. Also, given that the majority of fixed N is retained in soil organic matter and microbial pools, only a small fraction is actually transferred to the current-season rice crop. Microbial turnover rates, mineralization timing, and synchronization with plant N demand thus represent key bottlenecks linking ecosystem-level BNF to agronomic relevance. This section focuses on where and how much BNF occurs, whereas how management practices reshape these hotspots is addressed separately in Section 7.

In addition, temporal scaling remains a major challenge in quantifying BNF in paddy ecosystems. Nitrogenase activity and N₂ fixation rates can fluctuate substantially over short timescales in response to diel light cycles, transient redox shifts, fertilization events, and irrigation management. Consequently, BNF rates derived from short-term incubations or discrete growth stages should be interpreted as snapshots rather than season-averaged values. Robust estimation of seasonal BNF budgets will require repeated measurements across key phenological stages and management transitions, coupled with explicit integration of temporal variability.

## 5 Regulation of BNF by synthetic N inputs: suppression mechanisms and mitigation strategies

Synthetic N inputs exert a dual role in paddy systems: they are agronomically indispensable yet biologically suppressive to BNF. The inhibitory effects of synthetic N fertilizers on BNF have been confirmed by numerous studies (Tan et al., 2003; Jha et al., 2003; Smercina et al., 2019). This occurs because diazotrophs can acquire N not only from the atmosphere via BNF but also from externally available sources in the soil (Reed et al., 2011; Norman and Friesen, 2017). Given the high energetic cost of BNF, these microorganisms preferentially utilize external N inputs when available (Paul et al., 2000). This may also account for the phenomenon that diazotrophs with high N-fixing efficiency in pure culture often display much lower efficiency once inoculated into field soils, even when their survival is ensured. Pot experiments further confirmed this trend. Free-living N fixation tends to be higher in low-N environments than in N-rich conditions, whether in bulk soil, the rhizosphere, or moss ecosystems (Patra et al., 2006; Kox et al., 2016). Using ^15^N₂ tracer experiments, Zhang Y. H. et al. (2021) and found that at fertilization levels of 125–250 kg N ha^−1^, N₂ fixation rates declined by 81–86%. Interestingly, Teresita et al. (1986) reported that low fertilization levels (5.64 mg N kg^−1^ soil) did not suppress BNF, whereas higher application rates (99.72 mg N kg^−1^ soil) significantly inhibited it. A meta-analysis by Reis et al. across major Latin American biomes showed that experimental N addition reduced free-living BNF by an average of 30% (Reis et al., 2020).

**Figure 4:**
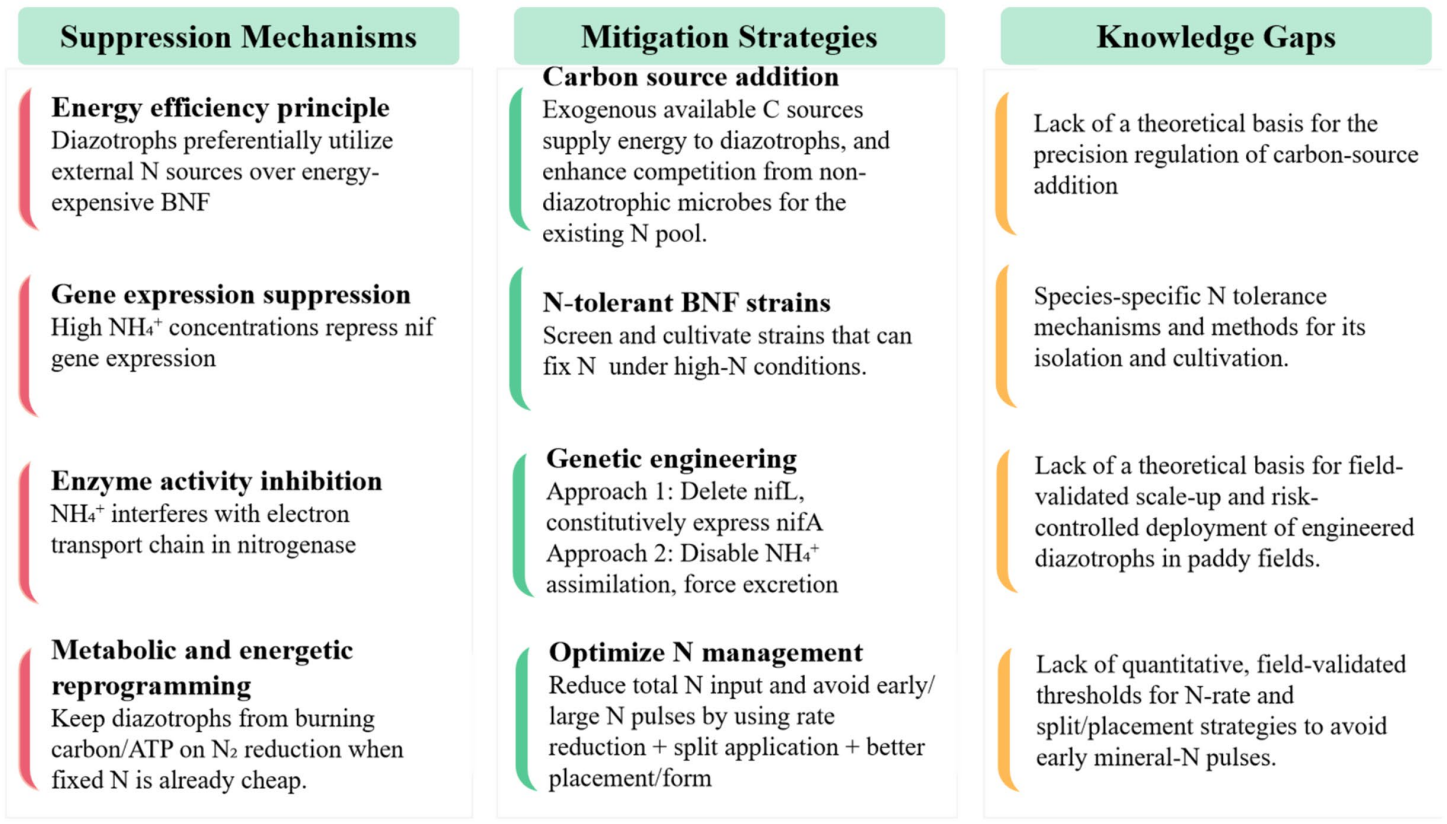
Regulation of BNF by synthetic N inputs: suppression mechanisms, mitigation strategies and knowledge gaps.

Significant progress has been made internationally in elucidating the mechanisms underlying this suppression effect (Figure 4): high concentrations of NH₄^+^ (>60 mg kg^−1^) inhibit nitrogenase activity by repressing nif gene expression and interfering with the electron transport chain (Reed et al., 2011). However, the addition of carbon (C) sources can, to some extent, alleviate the inhibitory effect of synthetic N fertilizers on BNF. Long-term straw incorporation has been shown to restore BNF activity by up to 68% (Tang et al., 2017). Laboratory experiments have shown that the application of 80 kg N ha^−1^ significantly reduced BNF, however, when 6 t ha^−1^ of straw was applied simultaneously, no reduction in BNF was observed (Charyulu et al., 1981). Based on a meta-analysis, Zheng et al. demonstrated that N addition suppresses BNF, but this inhibitory effect diminishes with increasing soil organic carbon (SOC), identifying SOC as a key regulator of N-fixation responses to N enrichment and terrestrial C-N coupling. Soluble C inputs stimulate the proliferation of heterotrophic diazotrophs, and straw with a high C/N ratio may promote heterotrophic bacterial N fixation more effectively than straw with a low C/N ratio (Naher et al., 2011; Zhang et al., 2022). At the same time, the application of soluble carbon substrates enhances microbial N immobilization, which can effectively reduce the soil ammonium concentration (Galloway et al., 2003), thereby alleviating its inhibition of nitrogenase activity and creating favorable conditions for BNF to proceed (Norman and Friesen, 2017; Zhang et al., 2022). In contrast, photosynthetic diazotrophs, such as cyanobacteria, exhibit limited responses to exogenous carbon inputs (Zhang et al., 2022). Nevertheless, the effects of different organic amendments on BNF vary substantially, for example, the incorporation of straw, farmyard manure, municipal organic waste compost, or biochar has been reported to exert markedly different impacts on BNF activity (Tanaka et al., 2006). At present, the quantitative relationships between the biochemical fingerprints of organic materials (e.g., C/N ratio, cellulose, and lignin contents) and the activation thresholds of BNF remain unresolved, resulting in a lack of theoretical basis for precision regulation. The molecular regulatory mechanisms of nitrogenase under carbon and N influences have been well reviewed in several studies (Batista and Dixon, 2019; Zhang W. et al., 2023; Gerhardt and Selim, 2025), therefore, we do not elaborate on them here. Despite these mechanistic insights, the quantitative relationships between the biochemical attributes of organic materials (e.g., C/N ratio, cellulose, and lignin contents) and the activation of BNF remain poorly resolved. Moreover, how these amendments alter soil habitat characteristics (e.g., microporosity, oxygen diffusion, water holding capacity) and, in turn, how these physical-microenvironments interact with diazotroph community composition and diversity to determine BNF rates are key unresolved questions.

N-tolerant diazotrophic inoculants hold promise for enhancing BNF under fertilization regimes. Different diazotrophic groups exhibit distinct responses of nitrogenase activity to exogenous N inputs (Smercina et al., 2019). For instance, N-tolerant diazotrophic strains such as *Pseudomonas stutzeri A1501* have been isolated, whose nif gene clusters harbor unique N regulatory elements (Yan et al., 2010). And the unicellular diazotrophic cyanobacterium *C. watsonii* maintained growth and fixation activity after two weeks of exposure to high nitrate concentrations (up to 800 μM) without inhibition (Großkopf and LaRoche, 2012). However, current research remains limited, as the discovery of resistant strains has largely relied on conventional culture-based methods, which suffer from high rates of strain omission due to the inability to target specific taxa. The use of ^15^N stable isotope probing (^15^N-DNA-SIP) in combination with high-throughput sequencing enables finer-scale resolution of active diazotrophs in paddy fields, providing insights into the identity, activity, and fate of BNF (Wang X. J. et al., 2020; Ma et al., 2019b). Research involving the applicant using this approach demonstrated that cyanobacteria represent the dominant diazotrophic group in paddy soils (Ma et al., 2019b). Therefore, applying ^15^N-DNA-SIP coupled with high-throughput sequencing to identify active diazotrophs under unfertilized (no N) and N-fertilized conditions maybe can inform targeted isolation strategies and guide the cultivation of N-tolerant strains for improved BNF.

Another potential strategy involves the genetic engineering of diazotrophs to disable their capacity to assimilate environmental ammonium, thereby forcing them to excrete ammonium ions instead. Bageshwar et al. (2017) found that engineered *Azotobacter chroococcum* HKD15, with the *nifL* gene deleted and *nifA* gene constitutively expressed, enhanced wheat yield by 60%, reduced urea use by 85 kg per hectare, and improved plant N content without affecting soil microbial populations. In *Azotobacter vinelandii*, combinations of mutations that deregulate nif expression (e.g., *nifL* loss-of-function) and attenuate ammonium reassimilation (via glutamine synthetase regulation or *amtB* transport) drive millimolar-level NH_4_^+^ excretion, and recent work has mapped genetic determinants enabling construction of strains with strong excretion while clarifying fitness trade-offs relevant to field use (Ambrosio et al., 2024). In associative bacteria more directly suited to cereals, *Azorhizobium caulinodans* has been rewired so that N_2_ fixation and NH_4_^+^ release are externally controllable (e.g., rhizopine-responsive circuits), minimizing metabolic burden and off-target N loss, an advance toward plant, directed N delivery (Haskett et al., 2022). Photosynthetic diazotrophs are also being developed: engineered *Rhodopseudomonas palustris* expressing Fe-only nitrogenase maintained activity under excess ammonium and acted as a biofertilizer that enhanced crop growth in tests, illustrating a complementary chassis for paddy ecosystems (Zeng et al., 2024). Together, these studies indicate that (i) relieving ammonium assimilation and transporter-mediated uptake, (ii) coupling nitrogenase activity to plant-derived signals, and (iii) selecting chassis adapted to flooded, redox-dynamic soils are key design principles for ammonium-excreting inoculants tailored to rice. Field-scale validation in true paddy systems, quantifying N transfer to rice, persistence/ competitiveness in complex communities, and containment of NH_3_ losses, remains the critical next step (Mus et al., 2022; Zeng et al., 2024). At the same time, the field application of engineered ammonium-excreting diazotrophs is constrained by ecological risks, which must be addressed to enable sustainable use in rice paddies.

## 6 The influence of other factors on BNF in paddy fields

**Figure 5:**
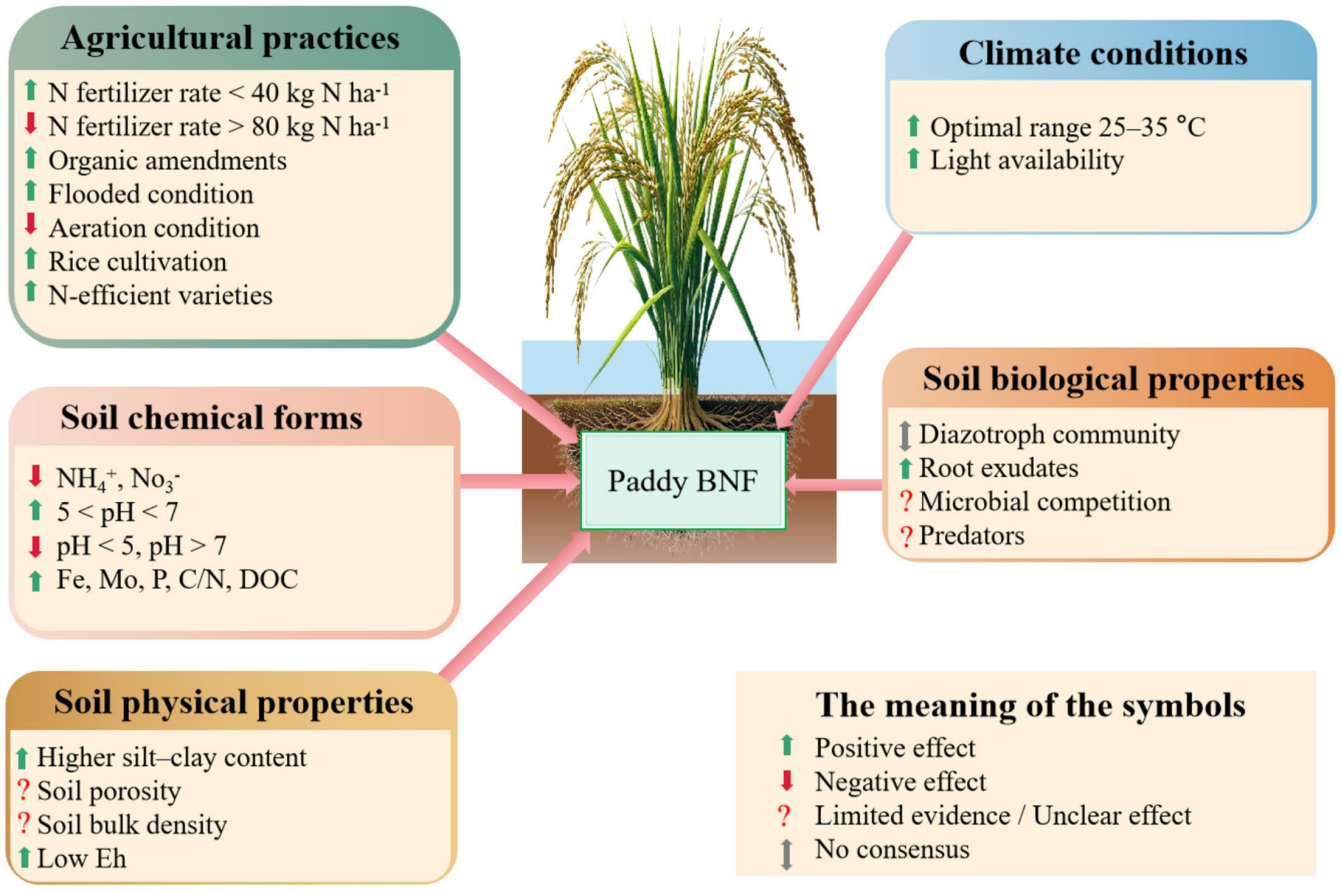
Soil physicochemical and biological properties, climate conditions, and farm management factors that affect paddy BNF.

In paddy field ecosystems, BNF is jointly regulated by multiple environmental and nutritional factors beyond N and C (Figure 5). Essential nutrients such as phosphorus (P), iron (Fe), and molybdenum (Mo) play pivotal roles in sustaining diazotrophic activity (Wang et al., 2018; Ma et al., 2019b; Yu et al., 2021). P is required for ATP biosynthesis, nucleic acid formation, and root-microbe interactions, and its deficiency markedly constrains nitrogenase function, whereas P fertilization has been shown to enhance BNF rates by supporting energy metabolism and stimulating rhizosphere processes (Wang et al., 2018). Iron constitutes the structural core of nitrogenase and key electron carriers, and its bioavailability in flooded soils is closely linked to redox dynamics that favor BNF under reducing conditions (Yu et al., 2021). Molybdenum, though needed only in trace amounts, is indispensable as the catalytic center of nitrogenase, with its availability often limited in acidic soils where it becomes immobilized. Using ^15^N₂ tracer experiments, in a rice-Inceptisol system under no N fertilizer input, the application of molybdenum (500 g sodium molybdate ha^−1^) markedly enhanced BNF, raising it from 22.3 to 53.1 kg N ha^−1^ (Ma et al., 2019b). Beyond nutrient availability, soil pH exerts a critical influence on diazotrophic community composition and nitrogenase activity, with near-neutral conditions generally favoring higher fixation rates compared with acidic soils (Wang et al., 2019), suggesting that liming or pH adjustment could enhance BNF in acidified paddy systems.

Soil water conditions, redox potential, and oxygen availability further shape the spatial and temporal dynamics of BNF. Flooding rapidly establishes reducing, low-oxygen conditions that protect nitrogenase, while midseason drainage or intermittent irrigation suppress fixation by increasing Eh and oxygen penetration, though re-flooding may induce compensatory surges in fixation activity. Under these fluctuating conditions, *cyanobacterial* and heterotrophic diazotrophs differ not only in their dominant habitats, illuminated surface biofilms/periphyton versus reduced microsites within aggregates and pores, but also in their underlying regulatory logic. *Cyanobacteria* reconcile oxygenic photosynthesis with oxygen-sensitive nitrogenase mainly through spatial separation (heterocysts that restrict O_2_ diffusion and enhance O_2_ scavenging) and/or temporal separation (dark-period fixation in non-heterocystous taxa), with ATP and reductant ultimately supplied by photosynthetic electron transport and carbohydrate transfer to N₂-fixing cells (Flores and Herrero, 2009; Reinhold-Hurek and Hurek, 1998; Magnuson, 2019). In contrast, heterotrophic diazotrophs are chiefly constrained by electron donor availability (labile C), respiratory demand, and access to micro-oxic/anoxic refugia; diffusion limitation inside structured matrices (biofilms/aggregates/pores) can create low-O_2_ microzones even when the surrounding bulk environment is oxic, enabling nitrogenase activity while maintaining external O_2_ exposure (Riemann et al., 2022). At the cellular level, some aerobic/microaerophilic heterotrophs further buffer transient O_2_ intrusions via high respiratory O_2_ consumption and protein-level “conformational protection” of nitrogenase (Narehood et al., 2025). Their electron-supply architecture can also rely on membrane redox pumps such as RNF complexes to generate the low-potential electrons required for nitrogenase under variable redox and substrate regimes (Zhang and Einsle, 2024). Together, these contrasts support a redox–light niche framework for paddy BNF in which photic–oxic surface layers favor cyanobacterial strategies (heterocysts, diel partitioning), whereas reduced microsites/aggregates and rhizosphere pockets favor heterotrophic diazotrophy supported by labile C and diffusion-limited O_2_; consequently, drainage-reflooding/AWD is expected to shift not only BNF magnitude but also the dominant diazotroph guilds and their key control points (Magnuson, 2019).

Rice cultivation and its specific genotype are also the factors in shaping diazotrophic communities and regulating BNF in paddy soils (Edwards et al., 2015). Bei et al. (2013) found that rice cultivation stimulated overall BNF and increased the dominance of heterotrophic over phototrophic BNF, with the ratio rising from 0.50 in fallow conditions to 0.99. Rice root exudates, which consist of a complex mixture of amino acids, sugars, and organic acids, provide essential carbon and N sources for N-fixing bacteria (Bürgmann et al., 2005; Knauth et al., 2005). Additionally, N uptake by rice plants reduces soil N concentrations, promoting heterotrophic N_2_ fixation around the roots (Singh and Singh, 1987). Research has shown notable variations in BNF across different rice varieties, with cultivated varieties exhibiting higher BNF than wild ones (Belnap, 2001; Ladha and Reddy, 2003). Ma et al. (2019a) found that BNF was measured at 22.3 kg ha^−1^ for inbred japonica W23 and 38.9 kg ha^−1^ for hybrid indica IIY, indicating that rice cultivar type exerts a substantial influence on the magnitude of BNF. Yan et al. (2022) further improved BNF in rice by genetically modifying the plants to increase the production of compounds like apigenin, which stimulate biofilm formation in N-fixing bacteria, promote bacterial colonization, and enhance BNF, ultimately leading to higher grain yields under N-limiting conditions.

Soil physical structure is an often overlooked but critical regulator of BNF in paddy soils. Features such as soil aggregation, pore size distribution, and diffusion constraints exert strong control over oxygen availability by shaping redox gradients and creating spatially heterogeneous microenvironments (Keiluweit et al, 2017; Riemann et al., 2022). These physical properties determine the degree of niche separation among diazotrophic communities, enabling oxygen-sensitive nitrogenase activity to persist within protected microsites even when surrounding conditions are oxic (Lam and Kuypers, 2011). In flooded paddy systems, diffusion limitation within aggregates and surface biofilms promotes the formation of low-oxygen microzones that favor heterotrophic and phototrophic diazotrophs, whereas in subsoil layers, pore connectivity and aggregate stability govern the persistence of anoxic refugia that support iron-reducing and other anaerobic N-fixing bacteria (Wang J. L. et al., 2020; Wang X. J. et al., 2020; Masuda et al., 2023). Soils of different textures (e.g., sandy, clayey, loamy) have inherent differences in aggregate stability, porosity, and gas conductivity. For instance, clayey soils tend to form finer, more diffusion-limited pore networks upon flooding, which may help maintain more extensive anaerobic microsites favorable for certain anaerobic diazotrophs (Moyano et al., 2012; Keiluweit et al., 2017). In contrast, rapid gas exchange in sandy soils may restrict the formation of such anaerobic niches. The resulting spatial heterogeneity in oxygen availability is a key driver of microbial community composition and activity, including diazotrophs (Liesack et al., 2000). Despite their fundamental role, soil physical attributes are rarely incorporated explicitly into conceptual or quantitative frameworks of BNF, which have traditionally emphasized chemical drivers and microbial community composition (Smercina et al., 2019; Reed et al., 2011). In reality, soil physical properties may interact with other factors to jointly regulate BNF. Zheng et al., using a meta-analytic approach, reported that higher soil bulk density and clay-loam content, despite theoretically favoring N fixation by increasing habitable soil space and creating low-oxygen conditions, could not fully account for the observed declines in N fixation. Although these properties enhanced N fixation responses under warming, drought, and multiple environmental stressors, they also explained 2–56% of the reductions in N fixation responses to warming, increased precipitation, and N deposition, indicating highly context-dependent effects driven by poorly understood mechanisms (Zheng et al., 2020). Recognizing soil physical structure as a co-determinant, alongside chemical and biological factors, of spatial BNF patterns is therefore essential for a mechanistic understanding of N fixation across surface and subsurface niches in paddy agroecosystems. The influence of soil physical structure on BNF in paddy soils remains insufficiently explored and warrants further investigation.

## 7 Integrated strategies for enhancing BNF in rice systems

The “C-N Synergy” Pathway: Optimizing the form of N fertilizer (e.g., controlled-release) combined with strategic organic carbon addition (e.g., straw). Agricultural practices primarily influence the process of BNF by modifying soil properties such as soil nutrient availability, microbial activity, and root exudation patterns. As discussed in Section 5, excessive mineral N suppresses BNF; therefore, optimizing fertilizer rate, timing, and placement may alleviate this inhibition. Interestingly, the inhibitory effect of chemical N fertilizers on BNF is stronger in subsurface soils (1–15 cm) than in the 0–1 cm surface layer, suggesting niche-specific differences in diazotrophic sensitivity to N suppression (Zhang Y. H. et al., 2021). This inhibition likely can be alleviated by optimizing fertilizer management (rate, timing, and placement) and by selecting enhanced-efficiency fertilizer types (e.g., controlled-release products and inhibitors) that reduce early/large mineral-N peaks, since controlled-release urea application and optimized N applied strategy can reduced soil NH_4_^+^-N, NO_3_^−^-N (Ren et al., 2022; Liu et al., 2023). However, these speculations require further experimental verification.

The “Plant-Microbe Partnership” Pathway: Coupling rice varieties with optimized root traits (exudation, O₂ release) with compatible, locally adapted microbial inoculants. Rice regulates BNF by mediating oxygen gradients through its roots, releasing photosynthates that fuel heterotrophic diazotrophs, and genetic variation among cultivars influences BNF by affecting root architecture, oxygen release, and exudate composition. Certain rice genotypes are better equipped to recruit and sustain beneficial diazotrophic communities, thus enhancing the BNF process (Knauth et al., 2005; Bei et al., 2013; Ma et al., 2019a). Different tillage practices, such as ridge tillage, no-tillage, and conventional moldboard plowing, exert distinct effects on diazotrophic communities in both the rhizosphere and bulk soil. For example, Hu et al. (2020) found that conventional moldboard plowing, compared with less intensive practices, reduces the relative abundance of free-living diazotrophs and disrupts both community diversity and network structure.

The “Water-Redox Steering” Pathway: Using water management (e.g., mild AWD) to dynamically shape oxic/anoxic interfaces and favor beneficial diazotroph guilds. Water management modulates oxygen availability and redox potential in rice soils, with continuous flooding favoring obligate anaerobes and phototrophic cyanobacteria. Although flooded paddy fields remain the dominant rice production system in Asia, water-saving or aerobic conditions, such as direct-seeded rice (DSR), alternate wetting and drying (AWD), semi-irrigated upland systems and fully aerobic “aerobic rice” systems, are becoming increasingly common (Nie et al., 2011; Anjali et al., 2025). Unless otherwise indicated, much of the classical BNF literature cited in this review derives from continuously flooded systems characterized by pronounced redox stratification, abundant phototrophic diazotrophs, and microaerophilic rhizosphere niches (Roger and Watanabe, 1996; Ladha and Reddy, 2003). However, emerging evidence shows that diazotroph community composition and BNF rates differ substantially under non-flooded or intermittently flooded conditions (Das et al., 2011; Zhang et al., 2025). AWD systems, for example, often show increased heterotrophic diazotroph abundance but reduced cyanobacterial activity; Rajaramamohan and Rao (1984) reported that nitrogenase activity increased as paddy soil shifted from dry to wet, but declined when it reverted from wet to dry. Similarly, Rao et al. (1983) found that rice rhizosphere nitrogenase activity was maximized under moderate water depth with small N and P inputs and soil-placed urea, but strongly suppressed by urea briquettes in shallow water and by prolonged deep flooding, highlighting the joint control of fertilization strategy and water regime on BNF. Compared with continuous flooding, intermittent irrigation may leave total BNF largely unchanged but redistribute fixed N from the surface and bulk soil to rice roots and shoots, likely via moisture-driven shifts in diazotroph communities (Zhang Y. H. et al., 2021). Straw incorporation under continuous flooding can more than double total BNF relative to intermittent flooding, primarily by increasing the abundance and activity of heterotrophic diazotrophs (e.g., *Desulfovibrio*, *Azonexus*, *Azotobacter*) (Zhang et al., 2025). Nevertheless, robust, comparable estimates of BNF across different water management regimes remain scarce, and incorporating findings from these increasingly widespread water-saving systems is essential for a realistic understanding of BNF potential in contemporary rice production.

Beyond their effects on BNF, agronomic practices also exert strong and often contrasting influences on greenhouse gas emissions, particularly nitrous oxide (N₂O) and methane (CH₄), in paddy ecosystems. Water management governs this trade-off: continuous flooding favors CH₄ production by sustaining anaerobic conditions, whereas alternate wetting and drying (AWD) markedly suppresses CH₄ emissions by introducing oxic phases, albeit often at the cost of enhanced N₂O emissions due to stimulated nitrification–denitrification processes (Yagi et al., 1996; Linquist et al., 2015). Fertilizer strategies further modulate these responses, as high mineral N inputs promote N₂O emissions, while optimized N rates, split application, and controlled-release fertilizers can reduce N₂O losses without yield penalties (Akiyama et al., 2010; Linquist et al., 2015). Organic amendments such as straw incorporation generally enhance CH₄ emissions under continuous flooding by supplying labile carbon, but their effects are attenuated under AWD or intermittent irrigation, with variable impacts on N₂O (Jiang et al., 2019; Wang H. et al., 2024). Collectively, these findings underscore the need for integrated management strategies that jointly consider BNF enhancement, crop productivity, and greenhouse gas mitigation, rather than addressing these objectives in isolation. Studies that jointly assess BNF and greenhouse gas emissions in paddy fields remain scarce, highlighting an important research gap for developing climate-smart and N-efficient rice systems.

## 8 Conclusion

This review synthesizes advances in BNF in rice systems and translates them into management implications. High mineral N inputs strongly suppress BNF, creating a paradox in which the very inputs used to secure high yields undermine the biological processes that could mitigate overuse. Recent *in situ* ^15^N_2_ studies highlight the disproportionate role of the top 0–1 cm soil layer, dominated by phototrophic cyanobacteria. However, whether these results are sufficient to reshape the classic rhizosphere-centric view and to identify surface niches as critical management targets still needs to be validated across a wider range of soil types and paddy systems.

For quantification, future work should improve long-term, in situ measurement under realistic field conditions and explicitly propagate methodological uncertainty into BNF budgets. Given that chronic fertilizer inputs may induce lasting legacy effects on diazotroph community composition, short-term “zero-N” assays are likely to substantially underestimate the attainable BNF potential, and the timescales over which diazotroph communities can be restructured remain poorly constrained. Addressing this knowledge gap will require multi-year, landscape-scale studies. Distinguishing between these causes, methodological discrepancies versus fertilization legacies, is critical for accurately interpreting the observed decline in BNF estimates and, consequently, for reassessing the agronomic and ecological significance of BNF in rice paddies.

On management, adding carbon (e.g., straw incorporation) can partially offset fertilizer-induced suppression, but BNF responses vary with the biochemical fingerprints of organic inputs (C/N, cellulose, lignin). Whether thresholds exist for the effects of N fertilizer application rate and carbon-to-N ratio on BNF, and what these thresholds might be, remains to be further studied. Selecting or engineering ammonium-tolerant or ammonium-excreting diazotrophs tailored to paddy niches is a promising route to sustain BNF under fertilization, provided that field-scale tests confirm competitiveness, N transfer efficiency, and environmental safety. Co-limiting factors, such as P, Fe, Mo, pH, and hydrological-redox dynamics, together with rice genotype effects (root oxygen loss, exudation) structure the magnitude and timing of BNF, underscoring the need for integrated fertilizer-water-variety-tillage optimization.

In sum, the transition toward low-input, environmentally sound rice production can proceed along two coordinated tracks: (1) Policy and practice, embed “less N, more C, water management, pH adjustment, targeted P/Fe/Mo” into technology packages; and (2) Microbial and crop innovations, including the development of N-tolerant or ammonium-excreting diazotrophic inoculants and rice cultivars that optimize plant-diazotroph interactions, provide a promising pathway to selectively enhance BNF and sustainability in paddy systems. Only by safeguarding yield while steadily raising the BNF share of crop N can we resolve the “N dilemma” and secure a durable N foundation for rice systems.
